# RBM14 as a novel epigenetic-activated tumor oncogene is implicated in the reprogramming of glycolysis in lung cancer

**DOI:** 10.1186/s12957-023-02928-8

**Published:** 2023-04-14

**Authors:** Yan Hu, Hanshuo Mu, Zhiping Deng

**Affiliations:** 1 Department of Respiratory, The First People’s Hospital of Zigong City, No.42, Shangyihao Road, Ziliujing District, Zigong City, 643000 Sichuan China; 2 Medical School, Nantong University, Nantong, 226001 Jiangsu China

**Keywords:** RBM14, Lung cancer, Epigenetic regulation, Glycolysis

## Abstract

**Background:**

RNA-binding motif protein 14 (RBM14) is upregulated in a variety of tumors. However, the expression and biological role of RBM14 in lung cancer remain unclear.

**Methods:**

Chromatin immunoprecipitation and PCR were carried out to measure the levels of sedimentary YY1, EP300, H3K9ac, and H3K27ac in the RBM14 promoter. Co-immunoprecipitation was used to verify the interaction between YY1 and EP300. Glycolysis was investigated according to glucose consumption, lactate production, and the extracellular acidification rate (ECAR).

**Results:**

RBM14 level is increased in lung adenocarcinoma (LUAD) cells. The increased RBM14 expression was correlated with TP53 mutation and individual cancer stages. A high level of RBM14 predicted a poorer overall survival of LUAD patients. The upregulated RBM14 in LUAD is induced by DNA methylation and histone acetylation. The transcription factor YY1 directly binds to EP300 and recruits EP300 to the promoter regions of RBM14, which further enhances H3K27 acetylation and promotes RBM14 expression. YY1-induced upregulation of RBM14 promoted cell growth and inhibited apoptosis by affecting the reprogramming of glycolysis.

**Conclusions:**

These results indicated that epigenetically activated RBM14 regulated growth and apoptosis by regulating the reprogramming of glycolysis and RBM14 may serve as a promising biomarker and therapeutic target for LUAD.

**Supplementary Information:**

The online version contains supplementary material available at 10.1186/s12957-023-02928-8.

## Background

Lung cancer remains the leading cause of malignant tumor-related deaths worldwide [1], with 85% of these cases being non-small cell lung cancer including lung adenocarcinomas (LUAD) and squamous cell carcinomas (LUSC) [2]. Although surgical resection has achieved good results in patients with early NSCLC, the total survival time of patients remains short. Most LUAD patients are diagnosed at an advanced stage with a low survival rate and high postoperative recurrence rate [3]. Therefore, it is necessary to uncover key regulatory molecules involved in the oncogenesis and progression of LUAD, which may lay the foundation for the development of new therapeutic strategies for patients with LUAD.

RNA-binding motif protein 14 (RBM14, also called CoAA) is amplified at chromosome 11q13 locus [4] and is reported to regulate transcription and alternative splicing [5, 6]. RBM14 is increased in a variety of tumors. The overexpression of RBM14 in NIH 3T3 cells promotes cell proliferation and colony formation [4], whereas the downregulation of RBM14 inhibits osteosarcoma cell growth [7]. Moreover, RBM14 enhances the transcriptional activity of PEA3 group member and is implicated in the migration of breast cancer [8]. RBM14 also is reported to increase radio-resistance of glioblastoma [9]. Besides, RBM14 can play the part of tumor suppressor by reducing the expression of c-Myc [10]. However, the effect of RBM14 on lung cancer remains unclear.

In the present study, we have explored the function of RBM14 and potential mechanisms in LUAD. Higher mRNA and protein level of RBM14 is found in LUAD tissues and cell lines. The upregulated RBM14 is caused by the DNA methylation and H3 acetylation of its promotor. The transcription factor YY1 directly binds to EP300 and recruits EP300 to the promoter regions of RBM14, which further promotes the expression of RBM14. YY1-induced RBM14 promotes cell growth and inhibits apoptosis by regulating the reprogramming of glycolysis.

## Methods

### Data resource and comprehensive analysis

Gene expression profiles were obtained from The Cancer Genome Atlas (TCGA) database (https://cancergenome.nih.gov/). Protein expression profiles were obtained from Clinical Proteomic Tumor Analysis Consortium (CPTAC) database (https://proteomic.datacommons.cancer.gov/pdc/). A summary of clinical data is shown in Supplementary Table S1-2.

### Cell culture

Human bronchial epithelial (HBE) cells and lung cancer cell lines (A549, PC9, H1299, and H1650) were obtained from ATCC. All cells were cultured in RPMI-1640 medium (Hyclone, Logan, USA) supplemented with 10% FBS (Gibco, Grand Island, USA) and antibiotics (100 units/ml penicillin and 100 μg/ml streptomycin) at 37°C in a humidified atmosphere (95% air, 5% CO_2_).

### Cell transfections

The specific siRNA of RBM14 and YY1 overexpressed vector were ordered from Sangon Biotech (Shanghai, China). The sequence of si-RBM14 is 5′-GCATTCTGGCCATAGAGCTCGTATT-3′. The sequence of the scrambled siRNA is 5′-TGCTGACTCCAAAGCTCTG-3′. After being incubated overnight, the siRNAs or plasmids were transfected by using lipofectamine 2000 reagent (ThermoFisher Scientific, Waltham, USA). Forty-eight hours after transfection, cells were harvested for further analysis.

### Reverse transcription-quantitative PCR

Total RNA was extracted using TRIzol® (Thermo Fisher Scientific). PrimeScript RT kit was used to reverse the mRNA into cDNA, and the SYBR-Green PCR Master One-Mix kit (TransGen, Biotech, Co., Ltd.) was used for quantitative real-time PCR. β-actin was used as an internal control. The sense and anti-sense primers were listed in Table 1.

**Table 1:** The primers used in this study

Gene	Sequences (5′-3′)	
	Sense	Anti-sense
RBM14	CTTCGACTACCAGCAGGCTTTT	CCGTCAGAGGCGCCACATAAG
EP300	ATTAAGGAACTGGAACAGGAG	AGAGGTCGTTAGATACATTGG
HK2	TGATGTGGCTGTGGATGAGCT	GCCAGGCAGTCACTCTCAATCTG
PDK1	GTGTAGATTAGAGGGATG	AAGGAATAGTGGGTTAGG
PKM2	GCACACCGTATTCAGCTCTG	TCCAGGAATGTGTCAGCCAT
LDHA	AGGCTGGGAGTTCACCCATTAAGC	GAGTCCAATAGCCCAGGATGTG
GLUT1	TGTCGTGTCGCTGTTTGTGGTGGA	TGAAGAACAGAACCAGGAGCACAG
GLUT3	GAGGTGCTGCTCACGTCTC	TTGAATTGCGCCTGCCAAAG
β-actin	ATTGGCAATGAGCGGTTC	CGTGGATGCCACAGGACT

### Chromatin immunoprecipitation assays

A Simple ChIP Enzymatic Chromatin IP Kit (#9002, Cell Signaling Technology, Danvers, USA) was used to evaluate the accumulation of YY1, EP300, H3K9ac, and H3K27ac in RBM14 promoter according to the manufacturer’s instructions. Briefly, after being crosslinked with EBM-2 containing 1% formaldehyde, the crosslinked cells were collected in a lysis buffer containing 1% PMSF. Chromatin was digested by micrococcal nuclease, and 2% of aliquots of lysate were used as input control. Lysates were incubated with 3 μg primary antibody or normal rabbit IgG, followed by immunoprecipitation with protein G agarose beads and incubation at 4 °C overnight with gentle shaking. DNA crosslink was reversed by the addition of 5 mol/L NaCl and Proteinase K at 65 °C for 2 h. Immunoprecipitated DNA was purified and amplified by PCR using specific primers. The IgG (ab172730, Abcam), anti-EP300 (ab275378, Abcam), anti-YY1 (ab109228, Abcam), anti-H3K9ac antibody (ab32129, Abcam), and anti-H3K27ac antibody (ab4729, Abcam) were used. Immunoprecipitated DNAs were analyzed by qPCR. Primer sequences targeting RBM14 promoter (−91~+7; ch11: 66616629-66616727, named RBM14-promoter) were as follows: sense, 5′-CATTCCTGAGGAGGACTGCC-3′ and anti-sense, 5′-TCTTCATTTTGTCGCCGCAG-3′.

### Western blotting

Cells were lysed by RIPA buffer (P0013C, Beyotime, Jiangsu, China), and the protein concentration was measured using a BCA kit (P0010, Beyotime). The protein was separated using SDS-PAGE and transferred onto a PVDF membrane (ISEQ00010, EMD Millipore). After blocking with 5% skimmed milk, the membrane was incubated with primary antibodies against RBM14 (ab70636, Abcam), YY1 (#63227, Cell Signaling Technology), p300 (#54062, Cell Signaling Technology), and β-actin (ab179467, Abcam) over-night at 4°C. After washing, the secondary antibody (Cell Signaling Technology) was added for 1 h of incubation. Protein signals were analyzed using an enhanced chemiluminescence substrate reagent kit (P0018M, Beyotime).

### Co-immunoprecipitation

Cells were lysed with IP buffer (1 mM EDTA, 20 mM HEPES, 150 mM NaCl, 0.05% sodium deoxycholate, and 0.05% NP-40) added protease inhibitors and phosphatase inhibitor cocktails. After centrifugation at 12,000 rpm for 10 min, the cell lysates were incubated with indicated antibody and protein A/G PLUS agarose beads (#9863, #37478, Cell Signaling Technology) overnight at 4 °C. After washing, the beads were lysed with loading buffer and boiled at 100 °C for 10 min. Then, the protein was detected by Western blotting.

### Cell Counting Kit-8

The transfected cells (1 × 10^4^ per well) were cultured in 96 well plates for 72 h of incubation. Then, 10 μl Cell Counting Kit (CCK)-8 solution (C0038, Beyotime) was added to each well and incubated at 37°C for 2 h. The absorbance was detected at 450 nm using a microplate reader.

### Apoptosis

Apoptotic cells were detected using an Annexin V-fluorescein isothiocyanate (FITC) Apoptosis Detection kit (#556547, BD Biosciences) according to the manufacturer’s instructions. Briefly, cells were re-suspended in 600 μl binding buffer. Then, 5 μl Annexin V/FITC and 5 μl propidium iodide (PI) were added. After being stained in dark for 15 min at room temperature, cells were analyzed by using a FACS Calibur (BD Biosciences).

### Glycolysis

Forty-eight hours after transfection, PC9 and A549 cells were cultured in the phenol red-free medium for 24 h. The level of glucose and lactate in the culture supernatant was detected by Glucose Assay Kit (#361510, Rsbio, Shanghai, China) and Lactate Assay Kit (#A019–2, Nanjing Jiancheng Bioengineering Institute, China) according to the manufacturer’s instructions. A Seahorse XF Glycolysis Stress Test kit (#103020-100, Agilent Technologies, Santa Clara, USA) was used to evaluate ECAR according to the manufacturer’s instructions. The basal ECAR was measured under the basal condition, followed by the sequential addition to each well of glucose (10 mM), oligomycin (2 mM), and 2-deoxyglucose (100 mM).

### Statistical analysis

All data analyses were performed using GraphPad Prism software (v6.02). Quantitative results are displayed as the mean ± standard error. Two-tailed Student’s *t* test and one-way analysis of variance (ANOVA) followed by Tukey’s test were used to compare the differences in two groups and multiple groups. Kaplan–Meier survival curve was analyzed with the log-rank test. Each experiment was conducted three times at a minimum. *P* < 0.05 was considered statistically significant.

## Results

### RBM14 shows higher levels in LUAD

**Fig. 1:**
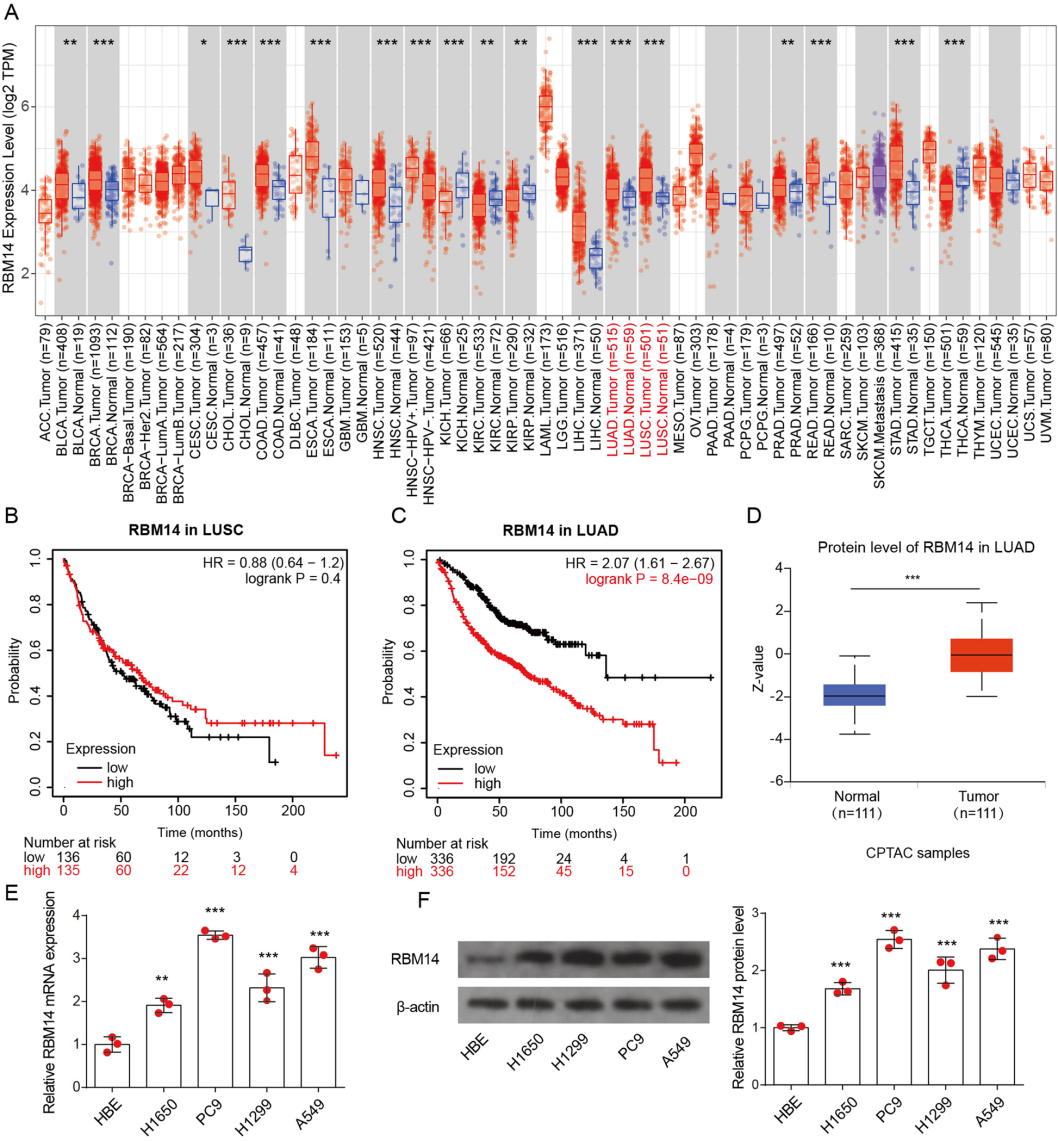
Increased RBM14 expression in LUAD. **A** RBM14 gene expression in various types of tumor tissues (red) and normal tissue (blue) was visualized by TIMER using TCGA dataset. **B**, **C** The prognostic curve of RBM14 was analyzed by Kaplan-Meier Plotter using TCGA LUSC or LUAD dataset. **D** RBM14 protein expression was visualized by UALCAN using CPTAC LUAD dataset. **E**, **F** RBM14 mRNA and protein level in HBE cells and lung cancer cell lines. **p <0.01, ***p <0.001. Three independent experiments were performed

We firstly assessed the level of RBM14 in various types of tumor tissues by Tumor Immune Estimation Resource (TIMER, cistrome.shinyapps.io/timer) database and found that RBM14 expression was significantly increased in BLCA, CESC, CHOL, COAD, HNSC, LICH, LUAD, LUSC, PRAD, READ, and STAD (Fig. 1A). Decreased RBM14 level was found in BRCA, KICH, KIRC, KIRP, and THCA. Here, we focused on exploring the function of RBM14 in lung cancer. Correlation analysis between RBM14 expression and prognostic value was assessed by the Kaplan-Meier Plotter database (Fig. 1B,C). A high expression of RBM14 in patients with LUAD was related to poor overall survival. However, in LUSC patients, the expression of RBM14 was uncorrelated to overall survival. Moreover, the protein level of RBM14 also was found to be upregulated in CPTAC LUAC samples (Fig. 1D). Besides, the mRNA and protein levels of RBM14 were significantly enhanced in lung cancer cell lines when compared with its levels in HBE cells (Fig. 1E, F). To reveal the relationship between RBM14 level and clinical-pathological parameters of LUAD patients, the UALCAN database was fully utilized. The data illustrated that RBM14 expression was closely associated with TP53 mutation status and individual cancer stage, whereas there was no relation between RBM14 expression and patient’s gender, patient’s age, patient’s smoking habits, and nodal metastasis status (Fig. S1). These results suggest that RBM14 is upregulated and significantly associated with the prognosis of LUAC.

### Effects of epigenetic regulation on RBM14 expression

**Fig. 2:**
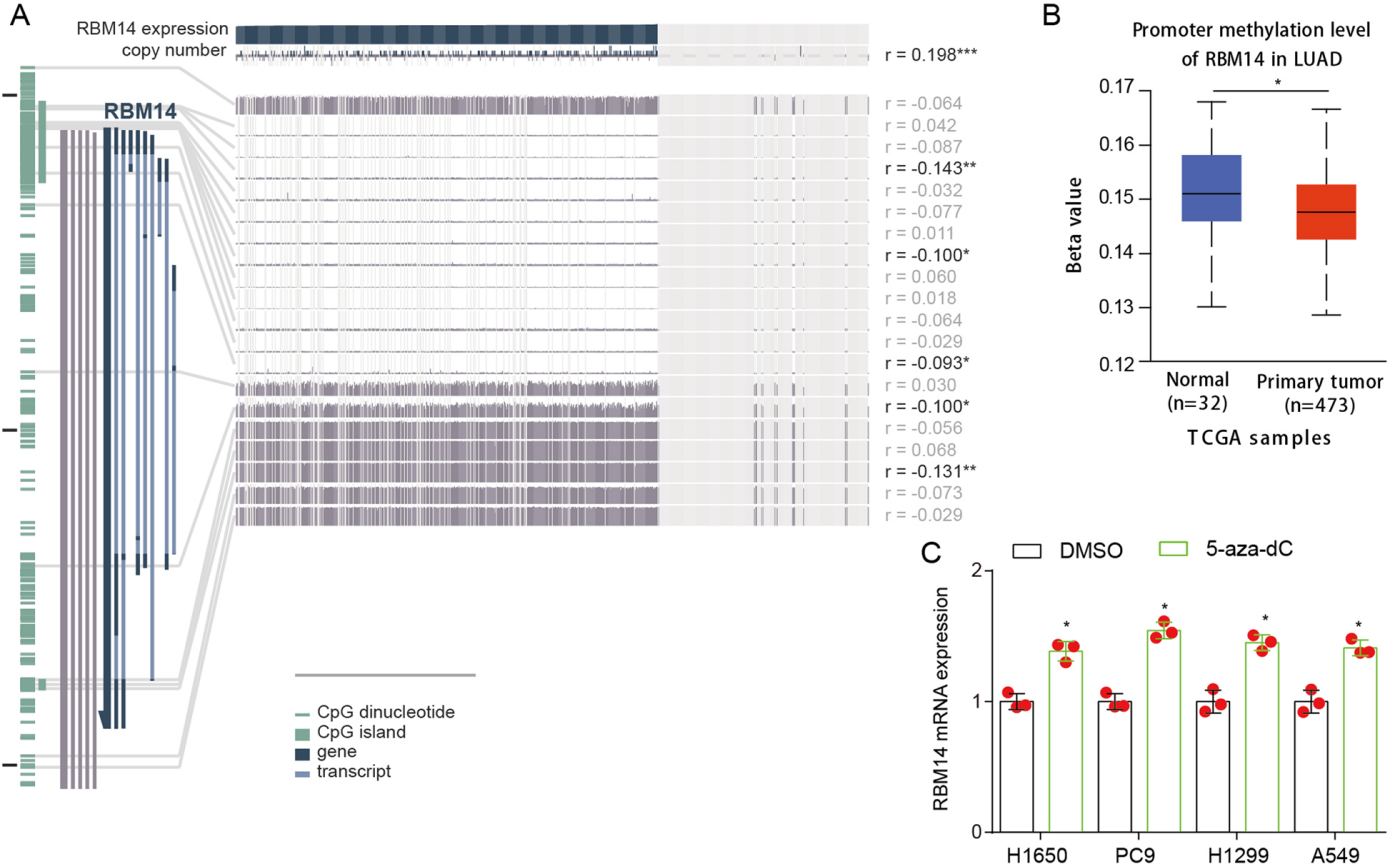
DNA methylation modifications the RBM14 promoter. **A** MEXPRESS view of the TCGA data for RBM14 gene in LUAD. **B** The promoter methylation level of RBM14 gene was analyzed by UALCAN using TCGA LUAD dataset. **C** The promoter methylation level of RBM14 gene in lung cancer cell lines after 5-aza-dC treatment. *p <0.05 vs DMSO. Three independent experiments were performed

Epigenetic regulation of promoters plays a very significant role in the expression of downstream genes. Considering that DNA methylation is an important mode of epigenetic modification, we explored the DNA methylation status of RBM14 promoter in the MEXPRESS database (Fig. 2A). The results indicated that methylation at CpG locations 66616247, 66616562, 66617965, 66624432, and 66626628 exhibited a statistically significant relationship with RBM14 expression. Besides, a significant association also was detected between RBM14 expression and copy number alteration. The data from UALCAN database suggested that the promoter methylation level of RBM14 in LUAD was significantly decreased (Fig. 2B). Moreover, the treatment of 5-aza-dC, an inhibitor for DNA methylation, slightly upregulated the level of RBM14 mRNA in four lung cancer cell lines (Fig. 2C), indicating that the expression of RBM14 was regulated by DNA methylation.

**Fig. 3:**
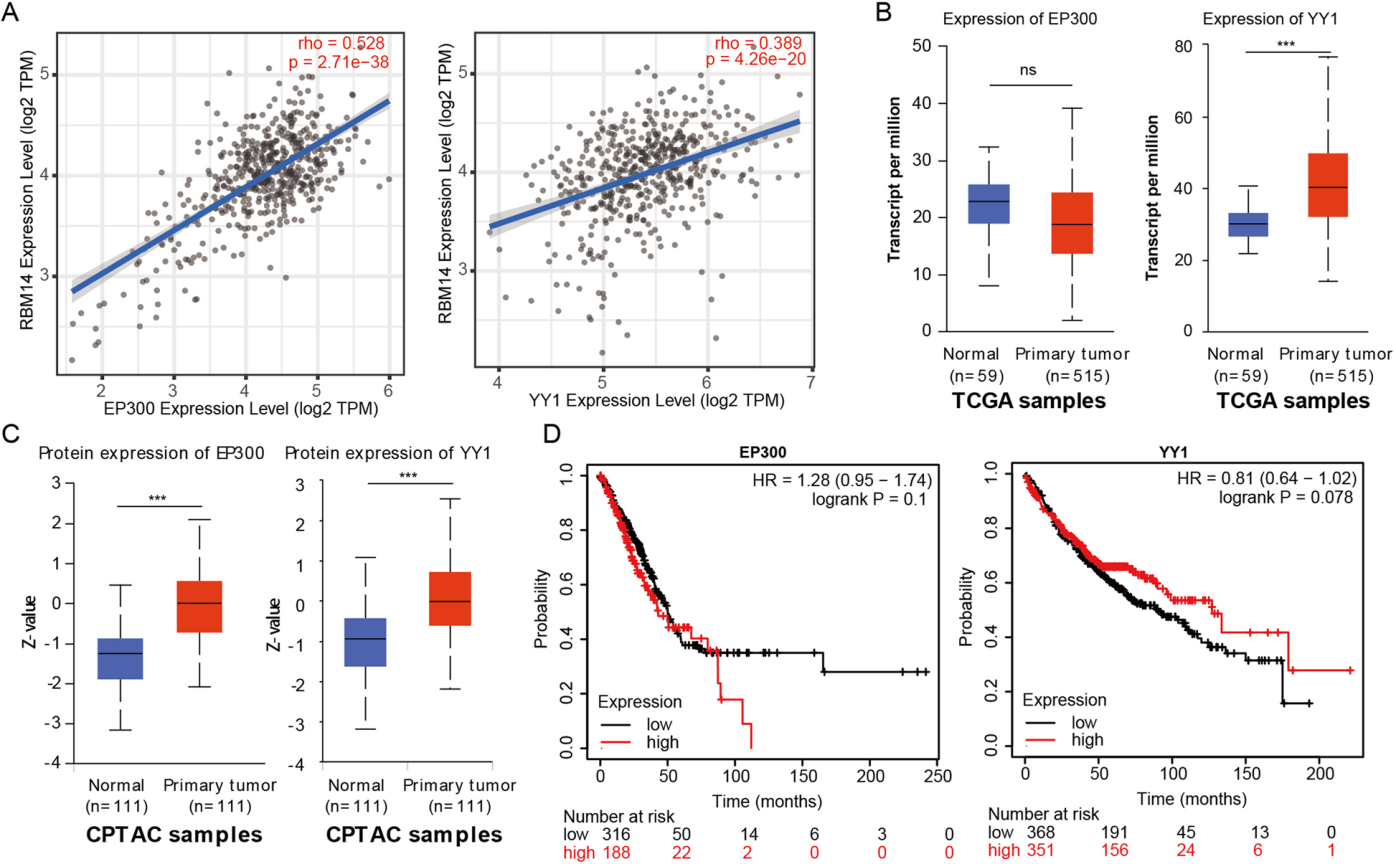
YY1 and EP300 expression showed a positive correlation with RBM14 expression in LUAD. **A** Correlation analysis of EP300 or YY1 expression with the level RBM14 was visualized by TIMER using TCGA LUAD dataset. **B** EP300 or YY1 mRNA expression in LUAD and normal tissues were visualized by UALCAN using TCGA LUAD dataset. **C** EP300 or YY1 protein levels in LUAD and normal tissues were visualized by UALCAN using CPTAC LUAD dataset. **D** OS survival curves for EP300 or YY1 in LUAD were analyzed by Kaplan-Meier Plotter using TCGA LUAD dataset. ***p <0.001. Three independent experiments were performed

Histone acetylation is an alternative mode of epigenetic regulation. EP300 is a histone acetyltransferase that not only possess acetyltransferase activity but is also able to bind to different transcription factors to form different transcriptional regulatory complexes that regulate the expression of target genes. By analyzing the ChIP-Seq data of A549 cells in ENCODE database, we found that there was an abundant accumulation of H3K9ac, H3K7ac, EP300, and YY1 in RBM14 promoter (Fig. S2A). Thus, we wondered whether the YY1-EP300 axis was implicated in the transcription of RBM14. By analyzing the association between RBM14 expression and EP300 and YY1 levels using TIMER database, we found that RBM14 expression was strongly associated with the expression of EP300, CBP, and CTCF in various types of tumor tissues (Fig. S2B). In LUAD tissues, RBM14 expression shows a positive correlation between the expression of EP300 and YY1 (Fig. 3A). LUAD tissues showed higher YY1 mRNA levels, but no significant change in the level of EP300 compared with normal tissues (Fig. 3B). It was worth noting that the protein level of EP300, and YY1 was increased in LUAD tissues (Fig. 3C). Besides, the survival curve showed that EP300 and YY1 cannot serve as an independent prognostic factor for LUAD (Fig. 3D). All these data indicated that the transcription of RBM14 may be regulated by YY1-EP300 axis-mediated histone acetylation.

### YY1 directly binds to EP300 and recruits EP300 to the promoter regions of RBM14

**Fig. 4:**
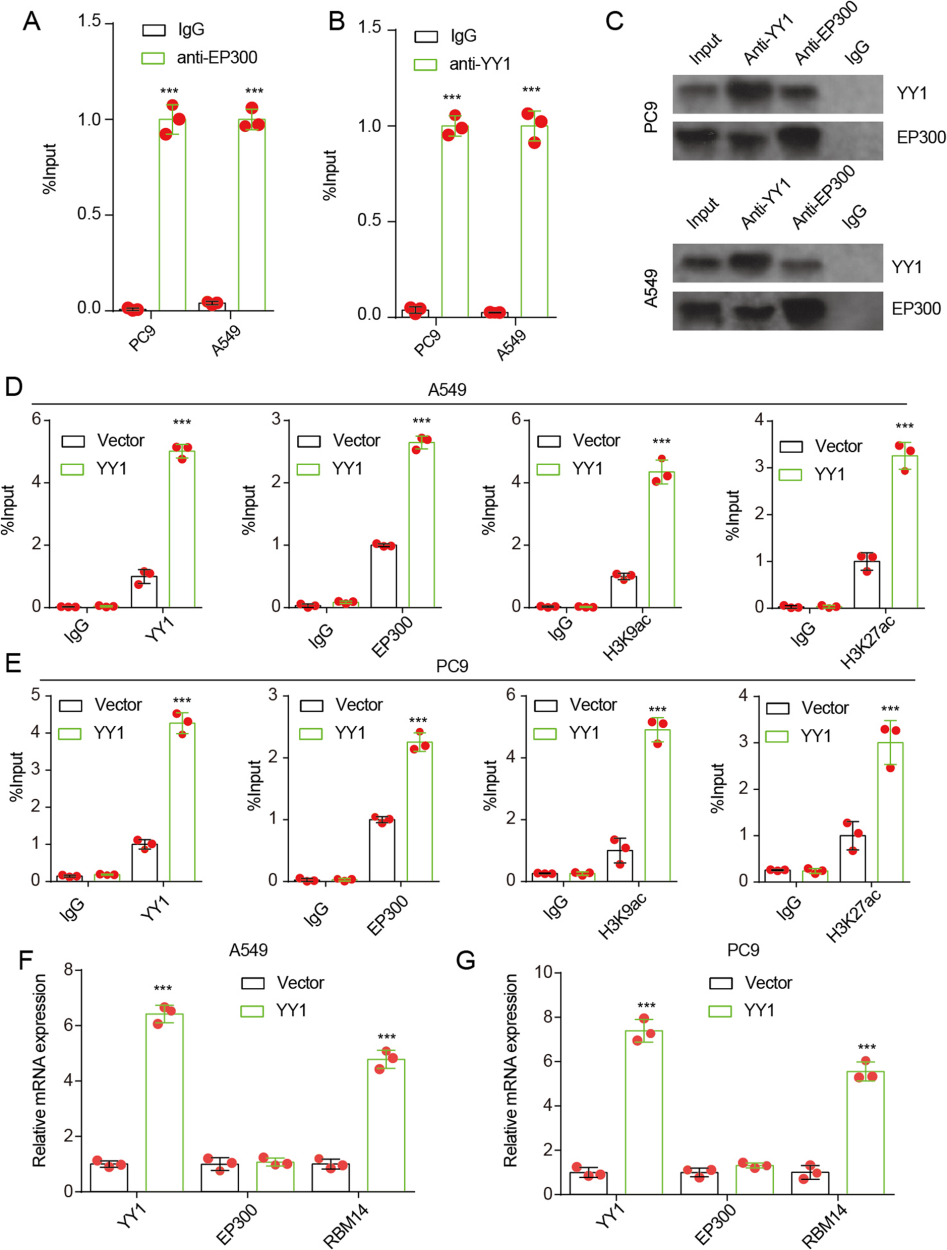
YY1 alters H3 acetylation in RBM14 promoter by recruiting to EP300. **A**, **B** ChIP assay was used to evaluate the accumulation of EP300 in RBM14 promoter. ***p<0.001 vs IgG. **C** The co-IP assay was used to affirm the interaction between EP300 and YY1 in PC9 and A549 cells. **D**, **E** ChIP assay was used to evaluate the accumulation of YY1, EP300, H3K9ac, and H3K27ac in RBM14 promoter after YY1 overexpression. ***p<0.001 vs Vector. **F**, **G** The mRNA level of YY1, EP300, and RBM14 in A549 and H1299 cells was detected after YY1 overexpression. ***p<0.001 vs vector. Three independent experiments were performed

The results of the ChIP assay also confirmed the accumulation of EP300 and YY1 in the promoter region of RBM14 (Fig. 4A,B). YY1 and EP300 can colocalize to the promoter region of RBM14, indicating potential interactions between YY1 and EP300 protein. To test this hypothesis, the co-IP assay was performed. The data indicated that there could be an interaction between YY1 and EP300 protein (Fig. 4C). Moreover, we found that YY1 overexpression increased the accumulation of YY1, EP300, H3K9ac, and H3K27ac in the promoter region of RBM14 in both PC9 and A549 cells (Fig. 4D-E). YY1 overexpression upregulated the mRNA level of YY1 and RBM14 but did not affect EP300 expression (Fig. 4F, G). These data suggest that YY1 overexpression enhances the recruitment of EP300, leading to the acetylation of H3 in the promoter regions of RBM14 and transcriptional activation of RBM14.

### YY1 promotes growth and inhibits apoptosis by epigenetic activation of RBM14 transcription

**Fig. 5:**
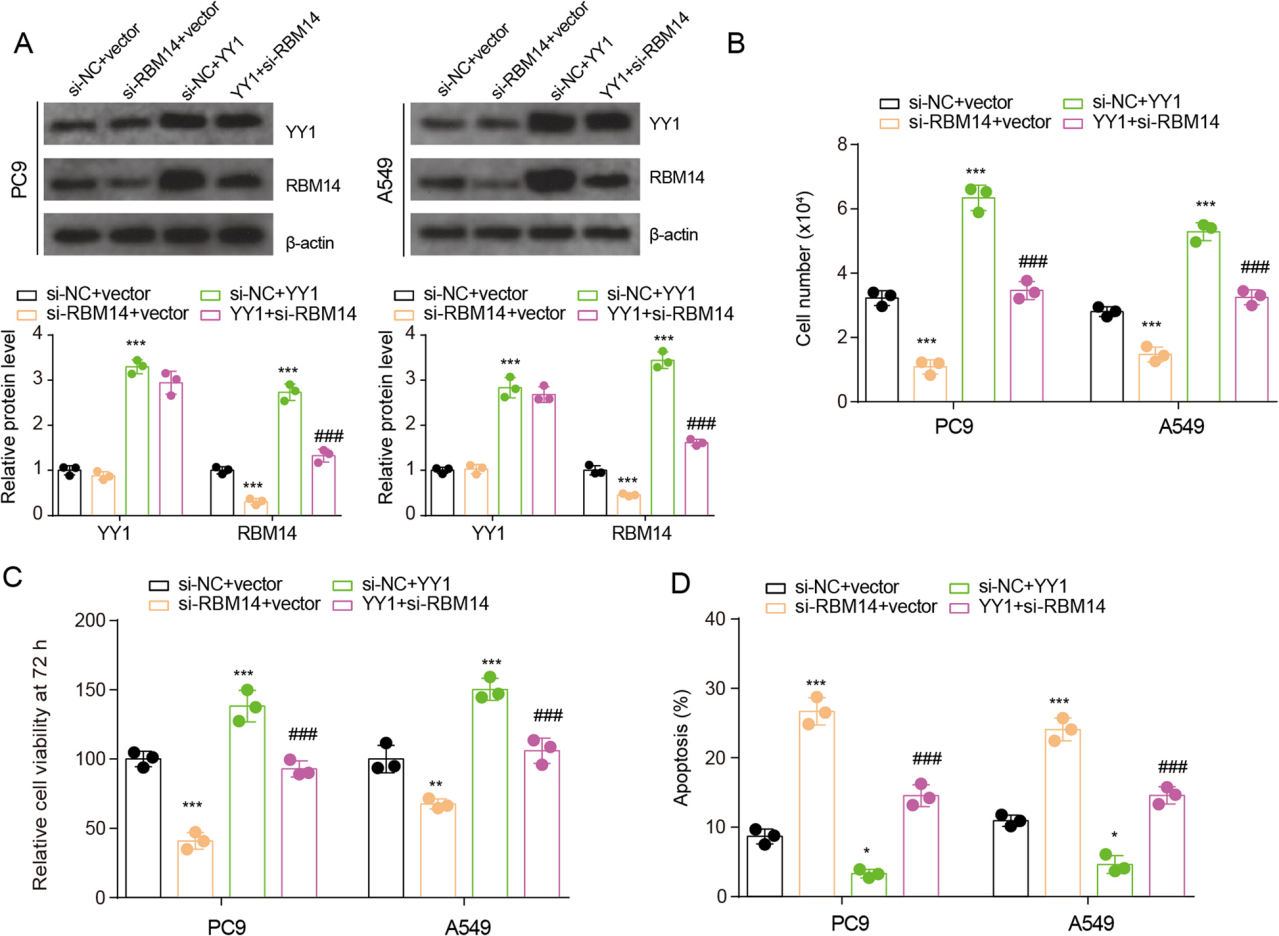
YY1 regulates growth and apoptosis by increasing RBM14 expression. **A** The protein level of YY1 and RBM14 in PC9 and A549 cells was detected by Western blotting in PC9 and A549 cells after YY1 overexpression or RBM14 knockdown. **B** Live-cell counting assay was used to evaluate the proliferation ability of PC9 and A549 cells after YY1 overexpression or RBM14 knockdown. **C** Cell viability of PC9 and A549 cells after YY1 overexpression or RBM14 knockdown was evaluated by CCK-8 assay. **D** The apoptosis of PC9 and A549 cells after YY1 overexpression or RBM14 knockdown was evaluated by flow cytometry. *p<0.05, **p<0.01, and ***p<0.001 vs si-NC+vector; ###*p*<0.001 vs si-NC+YY1. Three independent experiments were performed

We next explored the role of YY1-RBM14 on lung cancer cell growth and apoptosis. The specific siRNA for RBM14 (si-RBM14) decreased the protein level of RBM14 and did not affect the protein levels of YY1 (Fig. 5A). YY1 overexpression increased the protein level of YY1 and RBM14. Live-cell counting and CCK-8 assay displayed that YY1 overexpression significantly promoted the proliferation, whereas RBM14 knockdown inhibited the proliferation of PC9 and A549 cells (Fig. 5B-C). More apoptotic cells were present in RBM14 knockdown cells and lesser apoptotic cells were observed in YY1 upregulated cells (Fig. 5D). RBM14 knockdown reversed the change of growth and apoptosis in PC9 and A549 cells induced by YY1 overexpression. These results suggested that YY1-induced transcriptional activation of RBM14 is involved in the growth and apoptosis of lung cancer.

### The YY1-RBM14 axis is involved in glycolysis of lung cancer

**Fig. 6:**
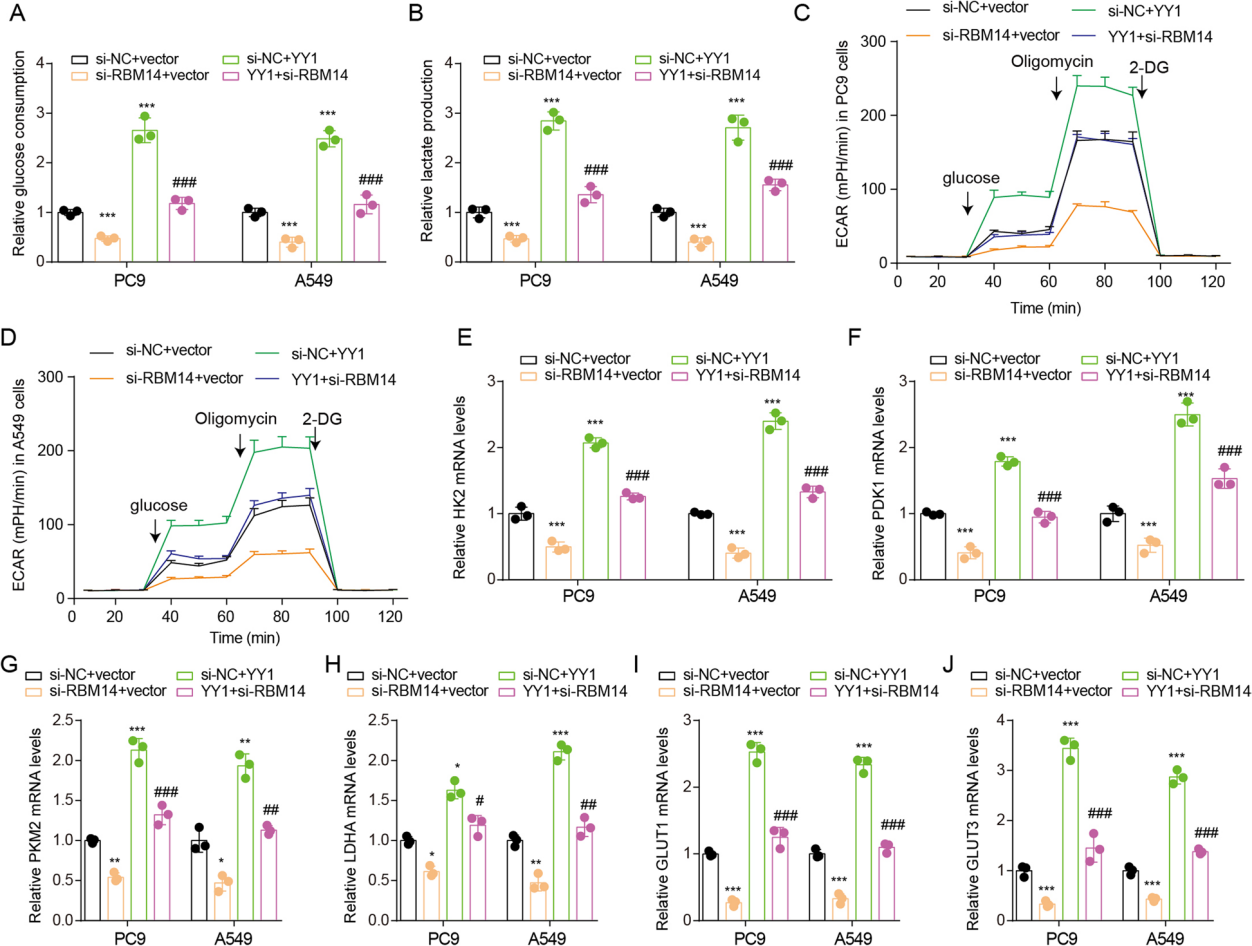
YY1 regulates glycolysis by increasing RBM14 expression. **A**–**D** Detection of glucose consumption, lactate production, and ECAR in PC9 and A549 cells after YY1 overexpression or RBM14 knockdown. **E**–**J** Detection of HK2, PDK1, PKM2, LDHA, GLUT1, and GLUT3 mRNA expression in PC9 and A549 cells after YY1 overexpression or RBM14 knockdown. *p<0.05, **p<0.01, ***p<0.001 vs si-NC+vector; ##*p*<0.01, ###*p*<0.001 vs si-NC+YY1. Three independent experiments were performed

The malignant proliferation of tumor cells depends on the energy supply of glycolysis. We then explored the function of the YY1-RBM14 axis on glycolysis. YY1 overexpression increased glucose consumption, lactate production, and ECAR in PC9 and A549 cells (Fig. 6A–D). However, RBM14 knockdown had the opposite effect. RBM14 knockdown rescued the enhanced glucose consumption, lactate production, and ECAR induced by YY1 overexpression. To further confirm the effect of the YY1-RBM14 axis on glycolysis, the glycolysis-related gene expression was assessed after YY1 overexpression or RBM14 knockdown (Fig. 6E–J). YY1 overexpression increased the mRNA level of HK2, PDK1, PKM2, LDHA, GLUT1, and GLUT3. RBM14 knockdown decreased the mRNA level of HK2, PDK1, PKM2, LDHA, GLUT1, and GLUT3 and recovered the effect on glycolysis-related gene expression mediated by YY1 overexpression. These results indicate that YY1 promotes glycolysis of lung cancer by enhancing RBM14 expression.

### The YY1-RBM14 axis is involved in immune infiltration in LUAD tissues

We also estimated the relationship between the YY1-RBM14 axis and immune infiltration by using TIMER database. High YY1 expression in LUAD tissues increased the infiltration of immune cells, such as CD8+ T cells, neutrophils, and macrophages (Fig. S3A). However, low YY1 expression in LUAD tissues increased the infiltration of B cells and Tregs. Besides, EP300 expression was positively related to the infiltration of CD4+ T cells, CD8+ T cells, Tregs, neutrophils, and macrophages (Fig. S3B). As shown in Fig. S3D, the expression of RBM14 had a positive correlation with the infiltration of CD4+ T cells, Tregs, and neutrophils. However, RBM14 expression did not correlate with the infiltration of CD8+ T cells, B cells, and macrophages. These results indicate a close association between the YY1-RBM14 axis and immune infiltration in LUAD.

## Discussion

There are only several studies suggesting that overexpression and deletion of RBM14 gene may be involved in tumorigenesis and progression, but the function and regulation mechanism of RBM14 in lung cancer remains unknown. Here, we identified RBM14 as an oncogene and the overall survival of patients with high RBM14 expression was significantly poor. The increased RBM14 is induced by DNA methylation and histone acetylation. RBM14 knockdown inhibited growth and promoted apoptosis by regulating glycolysis. Our result suggested that RBM14 can be a potential therapeutic target for lung cancer.

The occurrence and development of lung cancer result in the accumulation of genetic and epigenetic changes [11, 12]. DNA methylation, as the representative of epigenetic modifications, alters the expression of tumor-promoting genes or tumor suppressors [13–16]. Epigenetic silencing of MPDZ inhibits growth and progression of lung cancer [17] and epigenetic activation of FOXF1 confers cisplatin-resistant of NSCLC [18]. Here, we found that the DNA methylation levels of RBM14 promoter are decreased in LUAD and 5-aza-dC treatment promoted the expression of RBM14 in lung cancer cell lines, indicating that DNA methylation of RBM14 is one of the induction factors for the overexpressed RBM14 in LUAD.

Transcription factors can regulate gene expression by recruiting histone modification-related enzymes. YY1 functions as a transcriptional factor that could activate or restrain its target genes, depending on the cofactors that recruit it [19–21]. YY1 recruits EZH2 through its oncoprotein binding domain to inhibit gene expression by enhancing H3K27me3 [22]. Gao et al. indicate that YY1 interacts with spleen tyrosine kinase and inhibits SNAI2 transcription in lung cancer cells [23]. Wei et al. suggest that YY1 directly interacts with p300 and suppresses p53 stability, leading to the enhancement of cell proliferation and tumor growth [24]. KDM6A is recruited to the NTRK1 promoter by YY1 with subsequent enhancing NTRK1-encoded protein expression [25]. In this study, we confirmed the interaction between YY1 and EP300 and found that YY1 could promote RBM14 transcription by recruiting the HAT EP300, which transfers acetyl groups to histones.

YY1 has been reported to play crucial roles in various physiological functions, including cell proliferation, cell cycle, angiogenesis, metastasis, and glucose metabolism. Guo et al. find that YY1 participates in MIR31HG-mediated glycolysis colorectal cancer [26]. YY1 overexpression could reverse the decrease of glucose uptake, lactate production, ATP release, HK2, and LDHA proteins in circYY1 depletion breast cancer cells [27]. YY1 was also reported to promote the Warburg effect and tumorigenesis via glucose transporter GLUT3 [28]. Our data indicated that YY1 regulated glycolysis by promoting RBM14 expression.

## Conclusions

In summary, these results revealed that DNA methylation or YY1-EP300-mediated histone modification activates RBM14 transcription, which is implicated in the reprogramming of glycolysis in lung cancer. RBM14 is a potential target for treating lung cancers.

## Supplementary Information

**Additional file 1: Figure S1.** RBM14 gene expression level in different clinical trait subgroups was analyzed by UALCAN using TCGA LUAD dataset. **p <0.001. **Figure S2.** Epigenetic modification of the RBM14 promoter. (A) The enrichment of H3K9ac, H3K27ac, EP300, and YY1 in RBM14 promoter was evaluated by using ChIP-seq data of A549 cells in ENCODE database. (B) Correlation analysis of RBM14 expression with EP300 and YY1 expression was visualized by TIMER using TCGA dataset. **Figure S3.** Correlation of YY1-RBM14 axis with immune infiltration in LUAD. (A) Correlation analysis of YY1 expression with the level of immune cell infiltration in LUAD was visualized by TIMER. (B) Correlation analysis of EP300 expression with the level of immune cell infiltration in LUAD was visualized by TIMER. (C) Correlation analysis of RBM14 expression with the level of immune cell infiltration in LUAD was visualized by TIMER. **Supplementary Table S1.** The clinical information of lung adenocarcinoma samples in TCAG. **Supplementary Table S2.** The clinical information of lung adenocarcinoma samples in CPTAC.

**Additional file 2.**

## Data Availability

All data generated or analyzed during this study are available from the corresponding author on reasonable request.

## Abbreviations

RBM14: RNA-binding motif protein 14
ECAR: Extracellular acidification rate
LUSC: Lung squamous cell carcinomas
LUAD: Lung adenocarcinoma
TCGA: The Cancer Genome Atlas
CPTAC: Clinical Proteomic Tumor Analysis Consortium
HBE: Human bronchial epithelial
ANOVA: Analysis of variance
co-IP: Co-immunoprecipitation
CCK-8: Cell Counting Kit-8
