# Supplementary material for: RBM14 as a novel epigenetic-activated tumor oncogene is implicated in the reprogramming of glycolysis in lung cancer
Source: World J Surg Oncol. 2023 Apr 14;21:132. doi: 10.1186/s12957-023-02928-8 (PMC10105460; doi:10.1186/s12957-023-02928-8)
Supplement: Supplementary file 1 — Additional file 1: Figure S1. RBM14 gene expression level in different clinical trait subgroups was analyzed by UALCAN using TCGA LUAD dataset. **p <0.001. Figure S2. Epigenetic modification of the RBM14 promoter. (A) The enrichment of H3K9ac, H3K27ac, EP300, and YY1 in RBM14 promoter was evaluated by using ChIP-seq data of A549 cells in ENCODE database. (B) Correlation analysis of RBM14 expression with EP300 and YY1 expression was visualized by TIMER using TCGA dataset. Figure S3. Correlation of YY1-RBM14 axis with immune infiltration in LUAD. (A) Correlation analysis of YY1 expression with the level of immune cell infiltration in LUAD was visualized by TIMER. (B) Correlation analysis of EP300 expression with the level of immune cell infiltration in LUAD was visualized by TIMER. (C) Correlation analysis of RBM14 expression with the level of immune cell infiltration in LUAD was visualized by TIMER. Supplementary Table S1. The clinical information of lung adenocarcinoma samples in TCAG. Supplementary Table S2. The clinical information of lung adenocarcinoma samples in CPTAC. [file 12957_2023_2928_MOESM1_ESM.pdf]

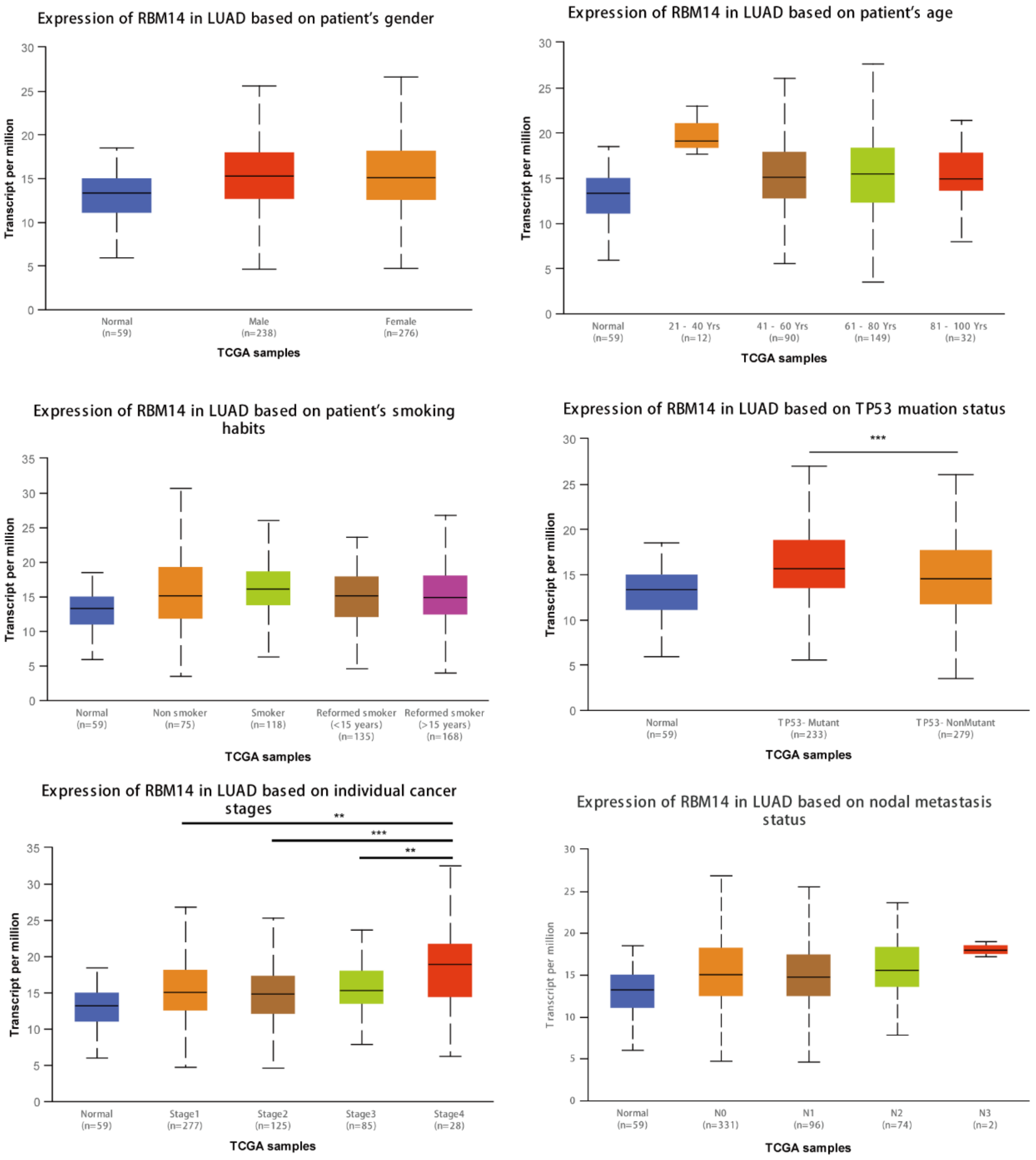

Figure S1. RBM14 gene expression level in different clinical trait subgroups was analyzed by UALCAN using TCGA LUAD dataset. **p <0.01, ***p <0.001.

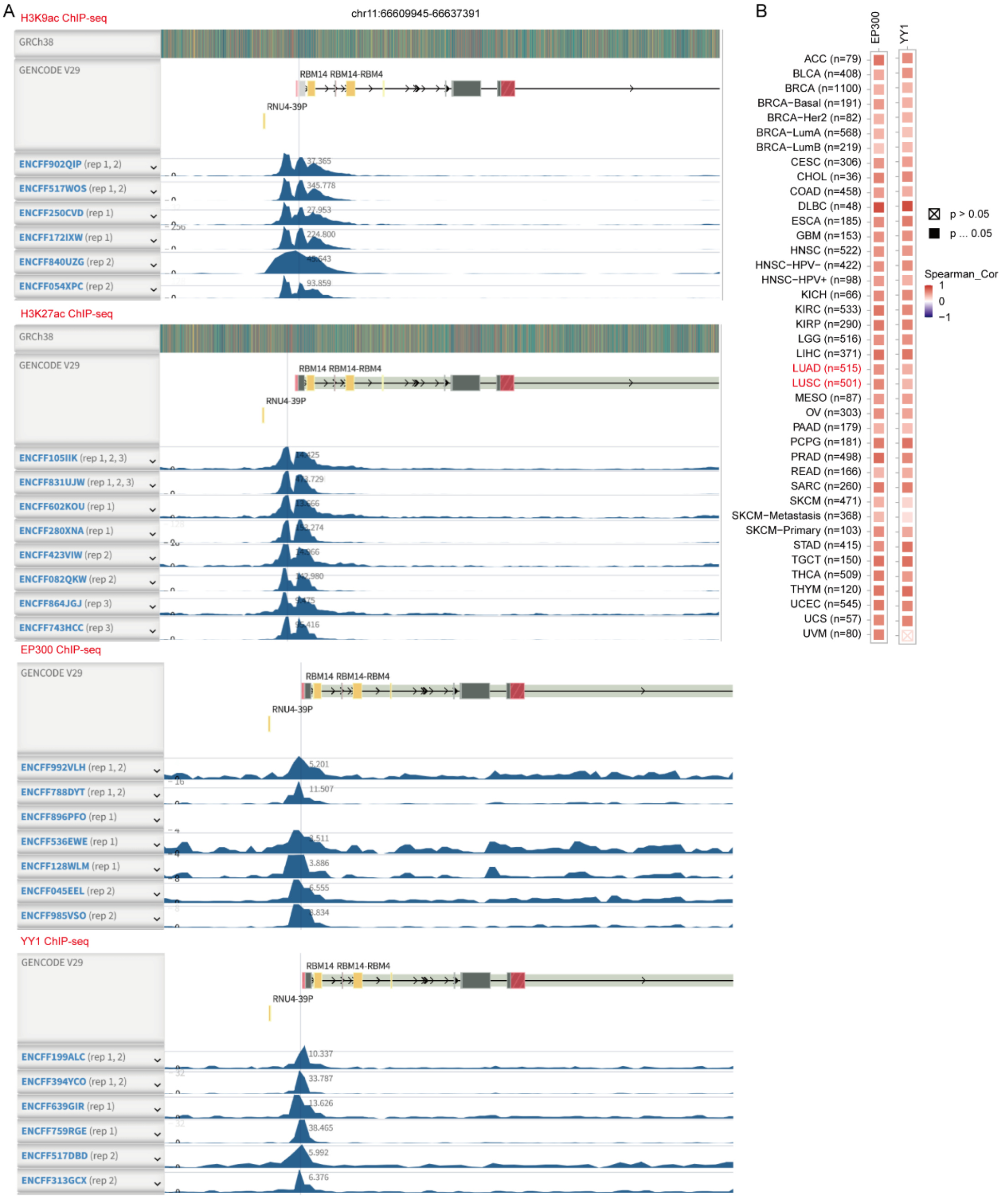

Figure S2. Epigenetic modification of the RBM14 promoter. (A) The enrichment of H3K9ac, H3K27ac, EP300, and YY1 in RBM14 promoter was evaluated by using ChIP-seq data of A549 cells in ENCODE database. (B) Correlation analysis of RBM14 expression with EP300 and YY1 expression was visualized by TIMER using TCGA dataset.

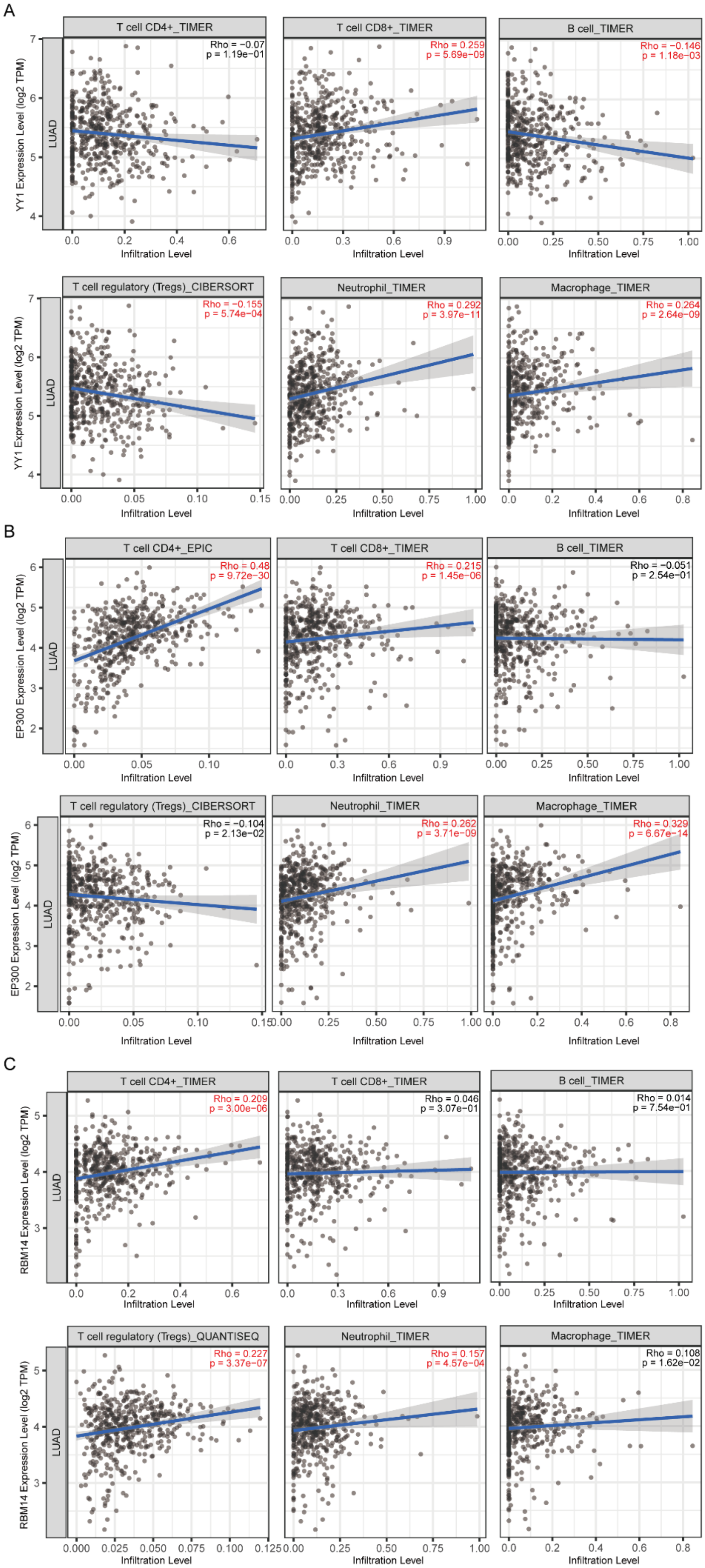
A
T cell CD4+_TIMER
Rho = −0.07
p = 1.19e−01
T cell CD8+_TIMER
Rho = 0.259
p = 5.69e−09
B cell_TIMER
Rho = −0.146
p = 1.18e−03
T cell regulatory (Tregs)_CIBERSORT
Rho = −0.155
p = 5.74e−04
Neutrophil_TIMER
Rho = 0.292
p = 3.97e−11
Macrophage_TIMER
Rho = 0.264
p = 2.64e−09
YY1 Expression Level (log2 TPM)
LUAD
Infiltration Level
B
T cell CD4+_EPIC
Rho = 0.48
p = 9.72e−30
T cell CD8+_TIMER
Rho = 0.215
p = 1.45e−06
B cell_TIMER
Rho = −0.051
p = 2.54e−01
T cell regulatory (Tregs)_CIBERSORT
Rho = −0.104
p = 2.13e−02
Neutrophil_TIMER
Rho = 0.262
p = 3.71e−09
Macrophage_TIMER
Rho = 0.329
p = 6.67e−14
EP300 Expression Level (log2 TPM)
LUAD
Infiltration Level
C
T cell CD4+_TIMER
Rho = 0.209
p = 3.00e−06
T cell CD8+_TIMER
Rho = 0.046
p = 3.07e−01
B cell_TIMER
Rho = 0.014
p = 7.54e−01
T cell regulatory (Tregs)_QUANTISEQ
Rho = 0.227
p = 3.37e−07
Neutrophil_TIMER
Rho = 0.157
p = 4.57e−04
Macrophage_TIMER
Rho = 0.108
p = 1.62e−02
RBM14 Expression Level (log2 TPM)
LUAD
Infiltration Level

Figure S3. Correlation of YY1-RBM14 axis with immune infiltration in LUAD. (A) Correlation analysis of YY1 expression with the level of immune cell infiltration in LUAD was visualized by TIMER. (B) Correlation analysis of EP300 expression with the level of immune cell infiltration in LUAD was visualized by TIMER. (C) Correlation analysis of RBM14 expression with the level of immune cell infiltration in LUAD was visualized by TIMER.

Supplementary Table S1 The clinical information of lung adenocarcinoma samples in TCAG

**Parameters**		Size
Age	<60	102
	>60	181
Gender	Male	238
	Female	276
Race	Caucasian	387
	African-American	51
	Asian	8
Individual cancer stage	Stage1/2	402
	Stage3/4	113
Smoking habit	Non smoker	75
	Smoker	118
	Reformed smoker	303
Nodal Metastasis status	No	331
	Yes	172

Supplementary Table S2 The clinical information of lung adenocarcinoma samples in CPTAC

**Parameters**		Size
Age	<60	48
	>60	63
Gender	Male	73
	Female	38
Race	Caucasian	34
	African-American	1
	Asian	1
Individual cancer stage	Stage1/2	89
	Stage3/4	31
