# Supplementary material for: RBM14 as a novel epigenetic-activated tumor oncogene is implicated in the reprogramming of glycolysis in lung cancer
Source: World J Surg Oncol. 2023 Apr 14;21:132. doi: 10.1186/s12957-023-02928-8 (PMC10105460; doi:10.1186/s12957-023-02928-8)

Fig. 1F

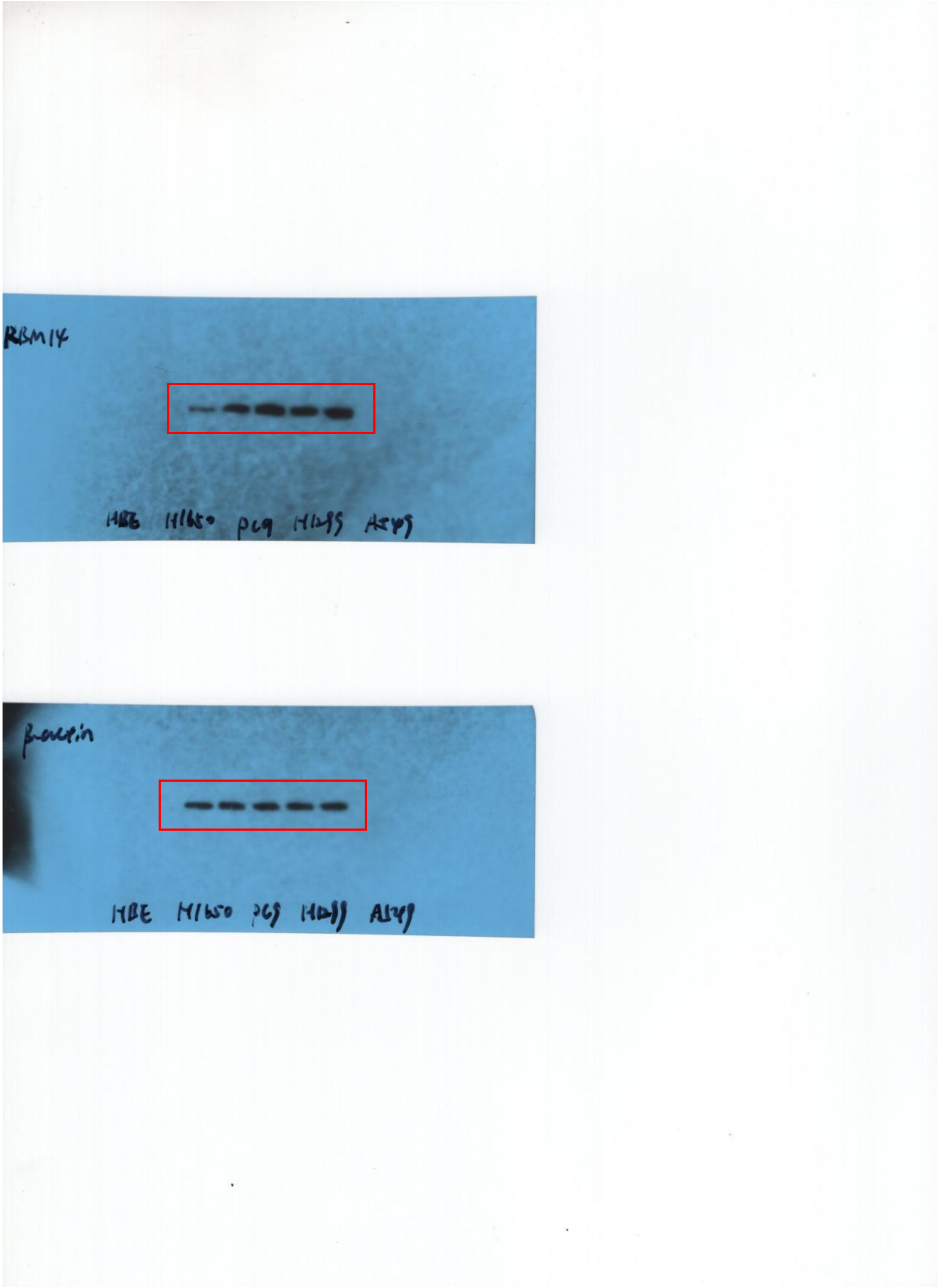

Fig. 4C_PC9

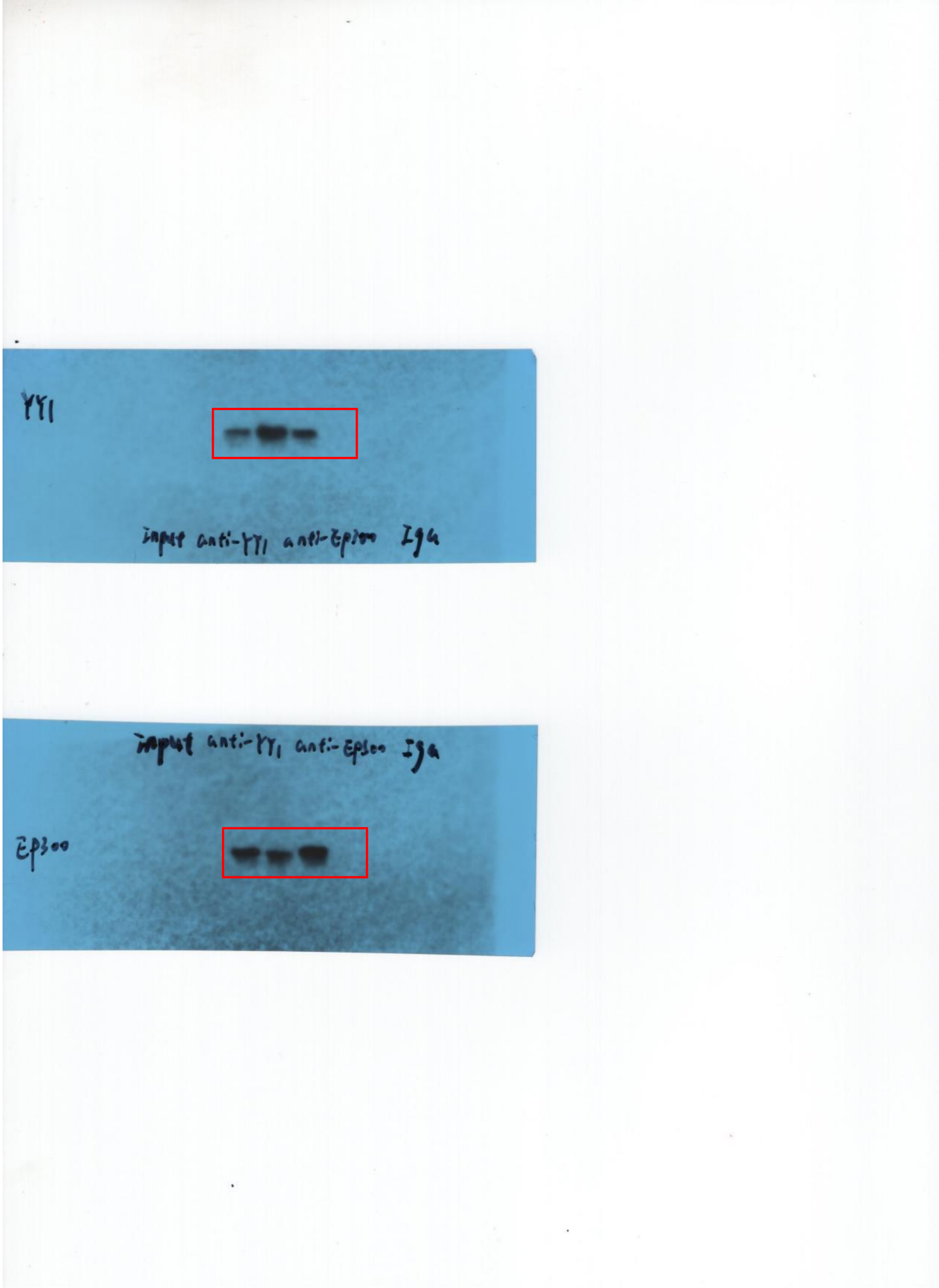
YY1
input anti-YY1 anti-Ep300 IgG
input anti-YY1 anti-Ep300 IgG
Ep300

Fig. 4C_A549

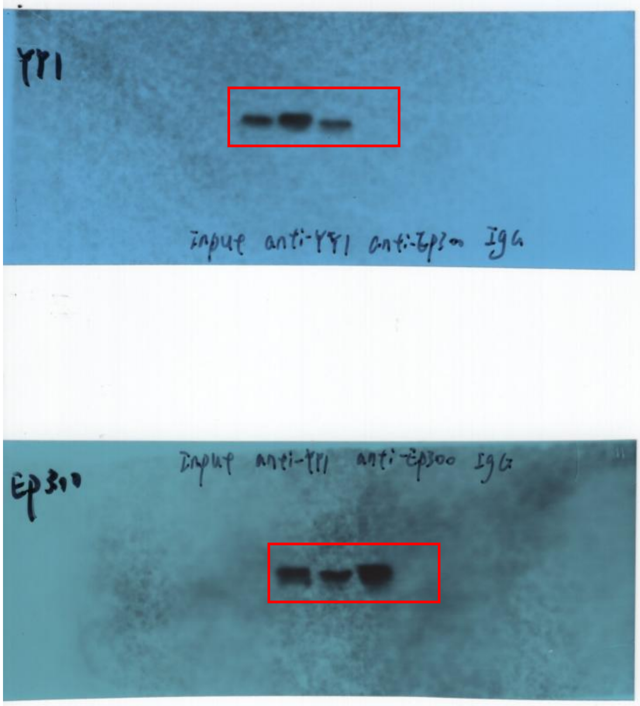
YY1
Input anti-YY1 anti-Ep300 IgG
Ep300
Input anti-YY1 anti-Ep300 IgG

Fig.5A_PC9

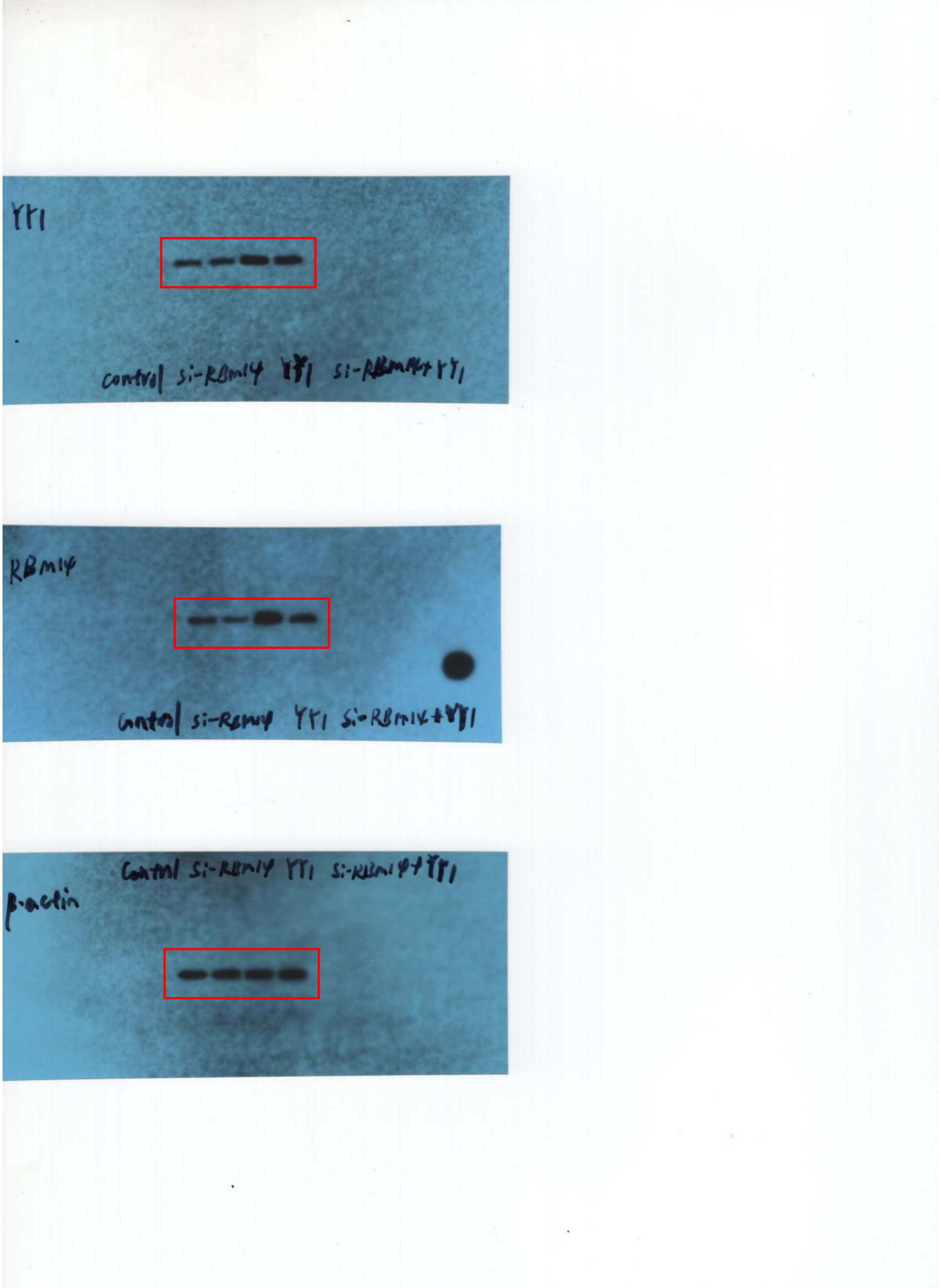
YY1
control si-RBM14 YY1 si-RBM14+YY1
RBM14
Control si-RBM14 YY1 si-RBM14+YY1
Control si-RBM14 YY1 si-RBM14+YY1
β-actin

Fig.5A_A549 (The group information is incorrectly marked. From left to right, control, si-RBM14, YY1, si-RBM14+YY1)

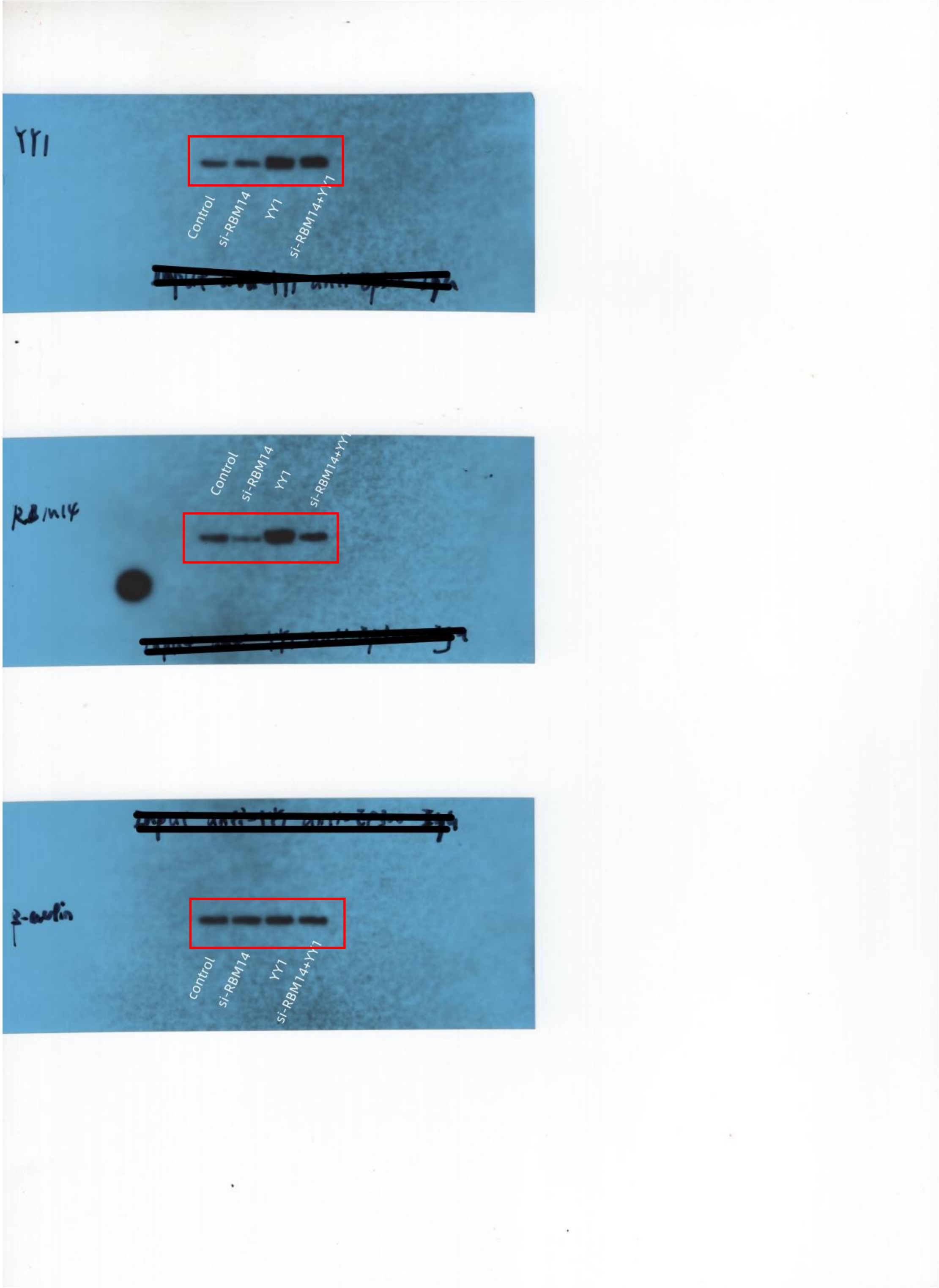

Supplement: Supplementary file 2 — Additional file 2. [file 12957_2023_2928_MOESM2_ESM.pdf]
